# Treatise on the Diseases of Women

**Published:** 1899-03

**Authors:** 


**Treatise on the Diseases of Women.** By ALEXANDER J. C. SKENE, M.D. Third Edition. Pp. xviii., 991. New York: D. Appleton and Company. 1898.

It is six years since the publication of the last edition of this work, and during that period great advances have been made, which have been duly incorporated in the present issue. The treatment of uterine fibroids has been fully brought up to date, but a more clear statement should have been made of the indications for adopting the different methods. The author believes that electrolysis "takes a high rank among the means of treating fibroma of the uterus," but we are not told the class of case in which he considers it desirable to use it. Too much space is given to the "illustrative cases," which, though interesting, occupy a greater part of the book than is warranted by the instruction they afford. The various subjects are handled with such thoroughness and judicious care as the great experience of the author would lead us to expect. A word of praise should also be given to the excellent plates and drawings with which the work is illustrated.

